# Open-access marine biodiversity records from the Colombia BIO Juradó-Cupica Expedition, Northern Colombian Pacific

**DOI:** 10.3897/BDJ.14.e189764

**Published:** 2026-06-24

**Authors:** Edgar Fernando Dorado-Roncancio, Erika Montoya-Cadavid, Armando Jose Rodríguez-Toscano, Luz Ángela López de Mesa, Luis Chasqui, Edgardo Londoño-Cruz, Rafael Mauricio Bechara-Escudero, Cristina Maria Cedeño-Posso, Eliana Barrios-Vásquez, Mayra Atencia-Galindo, Vanessa Yepes-Narvaez, Alejandro Rodríguez-Sánchez, Juliana Valentina Sánchez Muñoz, Nicoll Lizarazo, Natalia Ossa-Hernandez, Diana Valeria Gallego-Sánchez, Valeria Restrepo, Valentina Orozco Llanos, Hosmer Duvan Gomez-Vanega, Alexandra Paola Pardo Rivera, Laura Sofia González Carreño, Cristian Daniel Ortega Sánchez, Adriana Osorno-Arango, Shanly Coneo-Gómez, Luisa Fernanda Cera Marulanda, Jheyson José Mercado-Vides, Karen Sofia Murillo Jiménez, Harold Miguel Monge López, Sven Zea, Jose Tavera, Beatriz Susana Beltran-Leon, Yiliana Carolina Murillo Posso, Martha Patricia Vides Casado, David Alejandro Alonso Carvajal

**Affiliations:** 1 Instituto de Investigaciones Marinas y Costeras INVEMAR, Santa Marta, Colombia Instituto de Investigaciones Marinas y Costeras INVEMAR Santa Marta Colombia; 2 Universidad del Valle, Cali, Colombia Universidad del Valle Cali Colombia; 3 Universidad Tecnológica del Chocó, Quibdó, Colombia Universidad Tecnológica del Chocó Quibdó Colombia; 4 Universidad Nacional de Colombia, Santa Marta, Colombia Universidad Nacional de Colombia Santa Marta Colombia

**Keywords:** biological diversity, intertidal mudflats, mangrove, pelagic environment, rocky coastline, rocky reef, strategic ecosystems

## Abstract

**Background:**

The Instituto de Investigaciones Marinas y Costeras (INVEMAR), in partnership with the Universidad del Valle (UV), Universidad tecnológica del Chocó (UTCH) and the community councils of Cupica and Los Delfines, conducted the Colombia BIO Juradó-Cupica scientific expedition. This initiative, developed under the Colombia BIO strategy of the Ministry of Science, Technology and Innovation (Minciencias), intended to increase our knowledge about marine and coastal biodiversity associated with ecosystems between the township of Cupica and the municipality of Juradó, on Colombia’s northern Pacific coast. During the expedition, samples were collected from 34 stations located across five key ecosystems in the area: mangroves, a rocky coastline, intertidal mudflats, rocky reefs and the pelagic environment. As a result of observing and analysing specimens, the specialists were able to identify near 500 morphospecies including invertebrates, vertebrates, chormists and plants, belonging to 17 phyla. Some of these groups found numerous species that had not previously been detected in the north of the Colombia Pacific Region or even in tropical eastern Pacific Region. Similarly, the initiative makes an invaluable contribution to marine scientific collections by ensuring that the biological material obtained and its information are properly safeguarded, available and accessible, in order to continue promoting and strengthening marine research. The expedition also encouraged collaboration between national experts and local communities, facilitating the exchange of knowledge and incorporating traditional practices into the research process.

**New information:**

This study provides evidence of the presence of 468 species obtained from 2,107 records of observations, sightings and collected specimens from a single expedition, demonstrating a considerable taxonomic richness in the area. The number of new species records is particularly noteworthy, 96 for northern Chocó and 44 for the Colombian Pacific coast, particularly for Chordata, Annelida, Arthropoda, Cnidaria, Porifera and Chlorophyta. New distribution data acquired will be crucial in deepening our comprehension of species biogeography. The results significantly improve our understanding of marine and coastal biodiversity in northern Chocó, where information is scarce and emphasise the region's biological importance and its significant contribution to the Colombia's Pacific coast biodiversity, which is a region recognised as biodiversity hotspot. More broadly, the information expands national and regional marine biodiversity catalogues, enriching scientific collections providing input for future support of resource management and conservation processes in the region. Furthermore, from a social perspective, the data enable local communities to develop a better understanding of the ecosystems in their territory, thereby promoting the empowerment of local knowledge.

## Introduction

Scientific expeditions play a fundamental role in understanding ecosystems and their interactions, having evolved from initial incursions motivated by illegal extraction and scientific colonialism after the conquest to an interdisciplinary approach involving scientists from various fields, local experts and residents. This contemporary approach seeks to understand socio-ecosystems and the complex interaction that mutually sustains their subsistence (Maffi 2005, Berkes 2012). The initial explorations of the Colombian Pacific Region during the 16^th^-century explorations of Colombia's Pacific by Andagoya, Almagro and Pizarro targeted coasts and rivers (e.g. San Juan, Atrato), driven by the wealthy and Spanish expansion, but hindered by geographical and logistical limitations (Restrepo and Valencia 1994). The visit of Alexander von Humboldt and Aimé Bonpland (1799-1804), was a milestone in the development of systematic scientific expeditions in the region. These explorations boosted regional knowledge of biodiversity, focusing on the identification and description of the terrestrial flora and fauna of the Pacific (Humboldt 1982, Pardo Rojas and Escobar 2004). New contributions arrived from various international expeditions that took place between the early and mid-19^th^ century which sampled in the Colombian Pacific (Clark 1917, Fraser 1943, Hertlein and Strong 1955, Olsson 1971, NMNH - Smitsonian National Museum of Natural Histrory 2025).

Between the 70s and 90s, the Universidad del Valle and other institutions conducted several biodiversity and fisheries resource studies focusing on catchable and rocky areas of Gorgona Island, Utria Cove and Málaga Bay (Graham 1975, Birkeland et al. 1975, Cosel 1977, Cantera et al. 1979, Prahl et al. 1979, Robertson 1979, Glynn et al. 1982, Cosel 1984, Prahl 1986a, Prahl 1986b, Cantera et al. 1987, Cantera et al. 1989, Zapata et al. 1999a, Zapata et al. 1999b, amongst others). Subsequently, the advances in maritime technology and strengthened national capacity have enabled larger expeditions and more intensive studies of marine ecosystems, even in remote locations, such as Malpelo Island and other offshore areas (Invemar et al. 2006, Navas-Suárez et al. 2012, Invemar 2013, Comisión Colombiana del Océano 2018, Chasqui 2020, Invemar et al. 2022, National Geographic 2023). Data from these initiatives have been crucial for establishing protected areas and other conservation strategies (Ministerio de Medio Ambiente 2001, Cardique et al. 2016, Cruz 2021, Ministerio de Ambiente y Desarrollo Sostenible 2022).

Recognised as one of the world's 25 top biodiversity hotspots (Myers et al. 2000), due to its richness and endemism, the area has well-studied terrestrial biodiversity, but fragmentary marine knowledge. Research has focused on Punta Cruces and Cabo Marzo, with scarce data from remote northern coastal zones like Cupica and Juradó Bays near Panamá (Chasqui 2020). The area features diverse ecosystems, some strategically important to local communities and of high biological/natural significance. These include:

(1) Mangroves: provide habitats for reproduction, protection and feeding (Alongi 2002); and prevent coastal erosion (Barbier et al. 2011). Juradó features a large forest between Curiche and Partadó, medium ones in Estero Cacique and Punta Ardita and incipient stands elsewhere, totalling 2,237.1 ha, 5.4% of Chocoan Pacific mangrove (Eslava 1994, Velandia and Díaz 2016).

(2) Rocky coastline: coastal formations of unconsolidated soft rocks (clays, siltstones) and hard massive rocks (basalts, granite), with boulders, gravel and blocks. They feature steep slopes, the presence of sessile endobenthic communities that erode sediments, zoned epibenthic organisms and tidal pools with specific biological communities (Diaz et al. 2007). This ecosystem is found regularly in the study area between Cupica and Jurado.

(3) Intertidal mudflats: areas with fine sediments in low-to-moderate energy deltaic zones experience constant changes from sediment deposition, erosion and currents (Diaz et al. 2007). Featuring gentle slopes, they serve as resting and feeding grounds for migratory shorebirds. In the Tribugá and Cupica gulfs, they host rich invertebrate and demersal fish fauna.

(4) Rocky reefs (Riscales): submerged/emerged formations and small coastal islets (< 60 ha) vital for migratory seabird colonies (Diaz et al. 2007). Submerged zones support fish, algae, sponges, invertebrates and commercial species like snappers, groupers and tuna (Alzate et al. 2014). These reefs also function as aggregators of fish distributed along the coast, being more abundant in areas protruding from the coast, such as Cabo Marzo and Cabo Corrientes (Chasqui 2020).

(5) Pelagic environment: water column above seabed, including neritic zone (≤ 200 m over continental shelf) and oceanic zone. Pelagic species that live in mid-water or near the surface limit their contact with the seabed and coastline as much as possible and are classified in two ways: nekton (able to move independently regardless of currents) and plankton (organisms that cannot overcome ocean currents) (Diaz et al. 2007).

Due to scarce marine biological data in northern Chocó, the Colombia BIO Juradó-Cupica Expedition (11-27 Aug 2024) gathered biodiversity information on strategic marine and coastal ecosystems between Juradó and Bahía de Cupica municipalities. The expedition was led by INVEMAR, UV and UTCH, with funds from the Ministry of Science and Technology (Minciencias). Members of the Los Delfines and Cupica Community Councils participated in the expedition, joining the scientific team throughout the planning and execution process. It strengthened local capacities, exchanged traditional and scientific knowledge and promoted social appropriation. This manuscript presents the expedition contribution in terms of biological data to enrich the marine-coastal biodiversity inventories in this hotspot.

## Project description

### Title

Promotion of marine biodiversity research and social empowerment for the study of strategic ecosystems in the northern Pacific Region of Chocó; a baseline for resource inventory and sustainable use.

### Study area description

**Figure 1.**
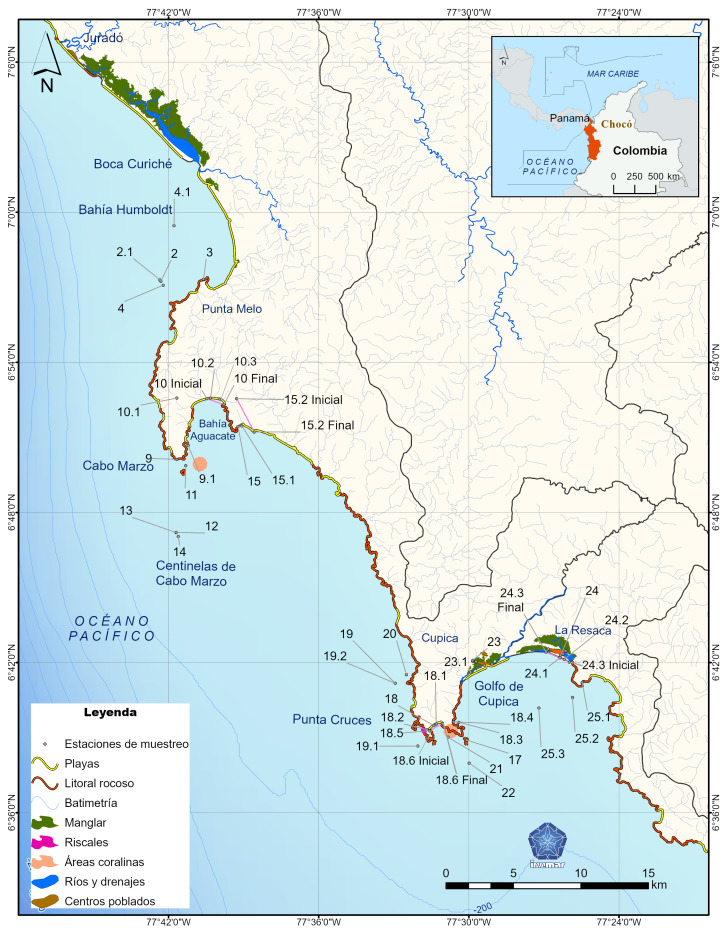
Location of stations sampled during the ‘Colombia BIO Juradó-Cupica Expedition’ in the northern Pacific Region of the Chocó Department.

This study focused on an area in the northern Colombian Department of Chocó, stretching from Punta de Chicocora in the south of Bahía Cupica to Humboldt Bay (Fig. 1). The region is described as a very humid tropical forest (Bmh-T) zone within the equatorial calm zone (Eslava 1994). The climate is influenced by the Intertropical Convergence Zone (ITCZ), generating local circulations and intense rainfall, mainly accentuated by orographic conditions (Velandia and Díaz 2016). Temperatures average 25-26.8°C. Chocó's North Pacific, one of the world's rainiest, exceeds 10,000 mm/year in spots (avg. 6,000 mm), with heavy rains early year, light Oct-Nov and up to 250 rainy days/year (3/4 days) (Cantera 1993, Eslava 1994). No dry season; lower precipitation Dec-Apr. The main tributaries in the Northern Region, under the jurisdiction of Juradó, are the Cupica, Octávida, Curiche, Putama, Partadó and Juradó rivers. In addition, mountain slope runoff creates numerous direct sea watercourses. These micro-basins/basins carry high water/sediment flows, depositing sediments that shape coastlines and seabed (Velandia and Díaz 2016).

### Design description

BIO Juradó-Cupica Expedition samples characterised ecosystems with 14 researchers (6 INVEMAR, 6 Univ. Valle, 2 UTCH). Sixteen locals from Curiché-Coredó, Cabo Marzo, Punta Piñas and Cupica aided daily sampling, guiding, transport and logistics. The BIO Juradó-Cupica Expedition (27 Jul-11 Aug 2024; 15 days) sampled fauna/flora for taxonomic inventories of key biological groups in Juradó-Cupica strategic ecosystems. The identification process used morphological analysis, DNA barcoding and regional reference catalogues/databases.

### Funding

Autonomous Heritage Resources National Fund for Science, Technology and Innovation 'Francisco José de Caldas', Contingent Recovery Financing Agreement No. 112721-421-2023, signed between MINCIENCIAS and INVEMAR, Code 107009. Co-financing Project 'Research for the generation and democratisation of knowledge, aimed at generating environmental justice, climate resilience, sustainable development and management of marine and coastal territories of national interest' Code BPIN 202300000000039.

## Sampling methods

### Study extent

Strategic ecosystems and sites were selected in consultation with local councils and scientists, taking into account traditional knowledge, location, natural features and landscape representativeness. Sampling was carried out at 34 stations (Fig. 1, Suppl. material 1) distributed across the selected strategic ecosystems: mangroves, rocky coasts, mudflats, rocky reefs and pelagic environments. Sampling involved intensive specimen search, recording or collection.

### Sampling description

Sampling was planned per ecosystem, defining methods, time (considering tides/location) and equipment for walking, snorkelling, diving or fixed-point observations.

### Quality control

1. During fieldwork, each visited ecosystem had an expert leader ensuring standardised collection, fixation and coding methods during fieldwork. The following data were included in the coded labels: sampling area; ecosystem origin; field station number; phylum identifier; unique consecutive number per taxon; and collection date. The coded samples were listed in Excel and verified against physical samples to prevent data duplication and to identify discrepancies affecting quality.

2. During processing, experts identified organisms using morphological and molecular analyses, assigning classifications and authorities from WoRMS catalogue (WoRMS Editorial Board 2025).

3. During documenting and preparing the data, the data tables were structured according to the guidelines of the Darwin Core Standard - DwC for occurrences data used by the Ocean Biodiversity Information System - OBIS (OBIS 2025a, OBIS 2025b), the Biodiversity Information System of Colombia - SiB Colombia (Plata et al. 2024) and the Global Biodiversity Information Facility - GBIF (GBIF 2025). The specific requirements of the BOLDSystems platform were also followed when entering the specimens' sequences and documenting their respective metadata (BoldSystems 2025).

4. During IPT publication process, the Colombian OBIS Node (SiBM - OBIS Colombia 2025) first perfomed the verification taking into account: (a) curatorial criteria (physical verification of lots and information); (b) taxonomic criteria (taxonomic validations); (c) spatial criteria (georeferencing validations); and (d) documentary criteria. The standardisation was then validated using different tools (e.g. databases, web services, applications and catalogues). Next, a double cross-check was performed, based on the established quality flags to ensure compliance with the requirements of OBIS (OBIS 2025c), SiB Colombia (Plata et al. 2024) and GBIF (GBIF 2025b), which are based on the Darwin Core Standard (TDWG 2025). Similarly, the completeness of the documented metadata was validated considering the guidelines of the Ecological Metadata Language Standard - EML (Jones et al. 2019). The dataset was validated once again through the Integrated Publishing Toolkit - IPT previously to the publication process.

### Step description

1. Preparation included reviewing weather/oceanographic conditions and safety meetings on locations, activities, component leaders, roles, objectives and safe sampling conditions per ecosystem.

2. Sampling in strategic ecosytems:

a. *Mangrove.* Sampling 1: Low-tide walking tours through mangroves (3-6 hours) involved intensive searches and manual collection of associated invertebrate and plant specimens (Fischer et al. 1995, Lazarus-Agudelo and Cantera-Kintz 2007), field labelling. Sampling 2: Fish associated with mangrove roots collected by netting at high-low tide transition, followed by review, collection (Castellanos-Galindo et al. 2006, Castellanos-Galindo and Villa 2008, Alzate et al. 2014), field labelling;

b. *Rocky coastline.* Intensive search and manual collection of organism-bearing loose rocks in supra-, meso- and infralittoral zones, using snorkelling or free diving where depth allowed (Londoño-Cruz and Cantera 2011). Relaxation with magnesium chloride (MgCl₂) and menthol crystals in seawater, preservation in 96% alcohol, field labelling;

c. Intertidal mudflats. Sampling 1: Macrofauna sampled directly with three corers (16 cm diameter x 20 cm depth) in supra-, meso- and infralittoral zones at low tide (Chapman 2007, Cantera et al. 2011, Cortés-Pineda et al. 2012). Relaxation with MgCl_2_ and preservation in 96% alcohol (Cortés-Pineda et al. 2012), field labelling. Sampling 2: Shorebird sightings: round trip along the beach, making specific observations of specimens, taking photographs, identifying and recording associated data (Lara et al. 2017);

d. *Rocky reef (Riscal).* Pre-dive safety meeting and equipment preparation. One-hour dive for intensive in situ organism search, manual collection; MgCl₂ relaxation and 96% alcohol preservation (Castellanos-Galindo et al. 2006, Chasqui 2020), field labelling. Recording of occurrences. Taking photographs when possible;

e. *Pelagic environment.* Sampling 1: Pelagic fish collected by handline/trolling; specimens photographed, tissue preserved in 96% alcohol, data recorded and field labelled. Sampling 2: Visual census of birds, mammals and reptiles during travel (opportunistic sampling), observations recorded 6 a.m. - 6 p.m. with hourly rest intervals (Lara et al. 2017). Photographs and data management. Sampling 3: Plankton sampling, 10-min horizontal surface trawling with 20/200/500-μm mesh nets (flow meter); fixed/preserved in 4% formalin neutralised with borax, field labelling (Boltovskoy 1981).

3. Separation, fixation and preservation of specimens and samples. The samples obtained were transferred to ship, inventoried and grouped by taxa. Specimens photographed; non-diagnostic tissue samples extracted for molecular analysis, labelled and preserved in absolute ethanol (periodically replaced) to maintain DNA viability (Barrero et al. 2008, Dorado-Roncancio and Arteaga-Flórez 2022). Finally, specimens were labelled and bagged for morphological taxonomic analysis in laboratory.

4. Taxonomic identification. Specimens were examined using standardised morphological approaches in the laboratory, high-resolution optics focusing on the key morphological structures for their specific identification, specialised keys and regional references (Terborgh and Winter 1982, Andrade 1993, Fischer et al. 1995, Myers et al. 2000, Hooper and Van Soest 2002, Brusca and Brusca 2003, Londoño-Cruz et al. 2011, Alzate et al. 2014, Zea et al. 2014, Chasqui et al. 2017, Lizarazo et al. 2020, Mejía-Quintero and Chasqui 2020, Mejía-Quintero et al. 2021, Robertson and Allen 2024, de Voogd et al. 2025). Infaunal samples were re-washed to remove fine sediments, sorted into major taxa (e.g. Annelida, Arthropoda, Mollusca, Echinodermata) and preserved in 96% ethanol. Photographic documentation was carried out using digital cameras, stereomicroscopes and optical microscopes (Zeiss Axio Lab. A1, Primo Star; Leica S6 D; Zeiss Axiocam ERc 5s). Measurements of key structures were made with ZEN Lite Blue 2.1 and LAS Leica software. Identifications targeted lowest taxonomic levels, primarily species, with morphotypes assigned when morphology was inconclusive. DNA barcoding amplified COI, 16S, 12S and 28S genes following INVEMAR protocols and Sanger-sequenced. Bioinformatics: sequence purification, alignment, > 500 bp consensuses, chromatogram curation and gap analysis for genetic variation (Hebert et al. 2003). The sequences obtained were compared with the genetic databases available in GeneBank (Sayers et al. 2025) and BoldSystems (Ratnasingham et al. 2024). Assignments: Species for > 99% identity; Genus for > 98% identity; higher categories for unmatched sequences (Kress and Erickson 2008).

5. Expert validation. The morphological identifications were compared with the molecular results, specifically for Annelida, Crustacea, Echinodermata, Mollusca, Platyhelminthes and fishes. Taxonomists resolved discrepancies between morphological and molecular approaches using expert judgement. The sequences in databases were validated and updated, creating the corresponding new species records.

6. Data and information structuring. Two Excel tables created, based on Darwin Core/SIBM-OBIS standards: 1) species occurrences (specimens, observations, sightings); 2) extended measures eMoF (sex, life stage, counts, behaviour) (OBIS 2025b, SiB Colombia 2025). BOLDSystems was used to document the molecular records and link sequences to data tables with a 'ProcessId' code.

7. Preparation and systematisation of metadata in IPT. Documentation of project information, participating entities and individuals, funding sources, temporal, taxonomic and geographic coverage, authors, bibliographic references and other data that enrich the context of the dataset. Validation against the EML metadata standard on the IPT platform.

8. Documentation and systematisation of molecular information. The sequences obtained by molecular methods were stored in BOLDSystems within the CCBIO container of Minciencias Colombia - Bio. All data and metadata were documented in accordance with the guidelines of this system.

9. Data quality controls. Four validation criteria were taken: (a) curatorial (physical verification); (b) taxonomic; (c) spatial (georeferencing); (d) documentary. DwC standardisation, in accordance with national guidelines for marine and coastal data, as well as the criteria required by global systems (OBiS and GBIF) and the OBiS Colombia and SiB Colombia. The following tools were used to optimise quality control processes during the application of each criteria. Taxonomic issues: Worms match taxa tool (https://www.marinespecies.org), Schmeyer´s Catalogue of Fishes of California Academy of Sciences https://www.calacademy.org/scientists/projects/eschmeyers-catalog-of-fishes, The World Porifera Database (https://www.marinespecies.org/porifera/). Spatial issues: https://data.canadensys.net/tools/coordinates, OBIS maptools (https://obis.shinyapps.io/coordinates/), Marine Regions Gazeteer https://www.marineregions.org/gazetteer.php?p=search, Google Earth Pro 2022 version 7.3.6.9345, Coding of Colombia's political-administrative division - Divipola (https://geoportal.dane.gov.co/geovisores/territorio/consultadivipola-division-politico-administrativa-de-colombia/). Controlled vocabularies: The NERC Vocabulary Server (https://vocab.nerc.ac.uk/collection), OBIS Measurement Types (https://mof.obis.org/). Validations: GBIF Data Validator (https://www.gbif.org/es/tools/data-validator), Mandatory elements for marine records – template for publishing biodiversity data from Colombia (https://biodiversidad.co/recursos/plantillas-dwc/#registros-biol%C3%B3gicos), OBIS Quality checks (https://github.com/iobis/obis-qc/), GBIF Data quality requirements: Occurrence datasets (https://www.gbif.org/data-quality-requirements-occurrences).

10. Publication of resources in OBIS and GBIF. OBIS Colombia verified OBIS/GBIF quality flags, mapped data/metadata in IPT, assigned DOI, registered in GBIF and published dataset to SIBM, SIB, OBIS and GBIF.

11. Deposit in scientific collections. The specimens and samples obtained were catalogued and stored in the following scientific collections: the Marine Natural History Museum of Colombia at Invemar (927 vouchers); the Hydrobiological Collection at the Universidad Tecnológica del Chocó (182 vouchers); and the Marine Biology Reference Collections at the Universidad del Valle (193 vouchers). Each collection issued certificates of deposit to provide official proof of compliance with national regulations.

## Geographic coverage

### Description

The records correspond to fauna/flora observations, sightings and collections from mangroves, rocky coastlines, mudflats, rocky reefs and pelagic zones between Cupica and Juradó, northern Chocó Pacific coast, from Cupica Bay (6°38'07.19"N; 77°27'18.64"W) to Curiché (7°01'25.55"N; 77°43'09.86"W). This area falls within the jurisdiction of the Los Delfines Community Council Collective (CCG Los Delfines) and the Cupica Community Council (CC de Cupica). Bathymetry: 0-42 m. Dataset includes extra-area biological records (bird/mammal sightings) from Buenaventura Port to sampling area, extending geographical coverage southwards (3.91474, -77.43464).

### Coordinates

6,991 and 3,915 Latitude; -77,424 and -77,726 Longitude.

## Taxonomic coverage

### Description

A total of 2,107 specimen records were obtained during the expedition. These represent 468 species, 493 genera, 337 families, 143 orders, 36 classes, 17 phyla and four kingdoms (Dorado-Roncancio et al. 2025).

Kingdom composition: Chromista - two phyla: Foraminifera, Ochrophyta. Plants - three phyla: Chlorophyta, Rhodophyta, Tracheophyta. Animalia invertebrates - 11 phyla: Annelida, Arthropoda, Brachiopoda, Bryozoa, Chaetognatha, Cnidaria, Echinodermata, Mollusca, Nemertea, Platyhelminthes, Porifera. Animalia chordates - Tunicata; Vertebrata: Chondrichthyes, Osteichthyes and Tetrapoda (birds, reptiles, mammals).

**Figure 2.**
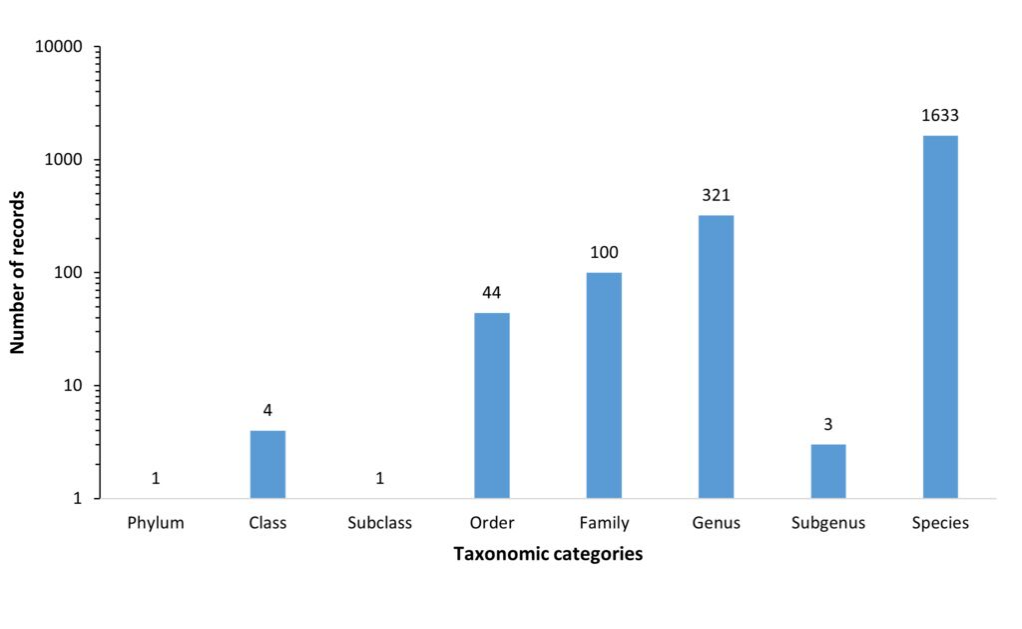
General scope of identification by taxonomic categories.

**Figure 3.**
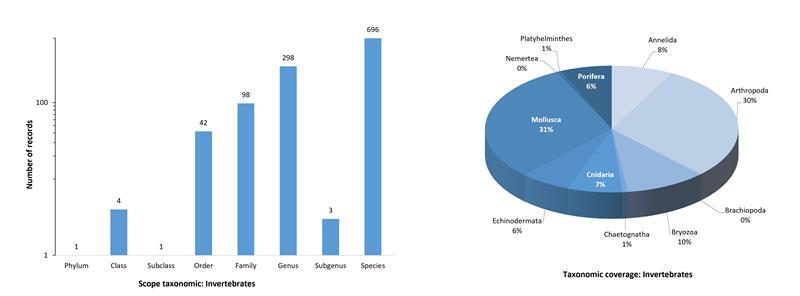
Scope of taxonomic identification for invertebrates. Left: Scope by taxonomic categories considering the total number of records. Right: Taxonomic coverage by phyla considering only the specimens proporcion identified to species level.

**Figure 4.**
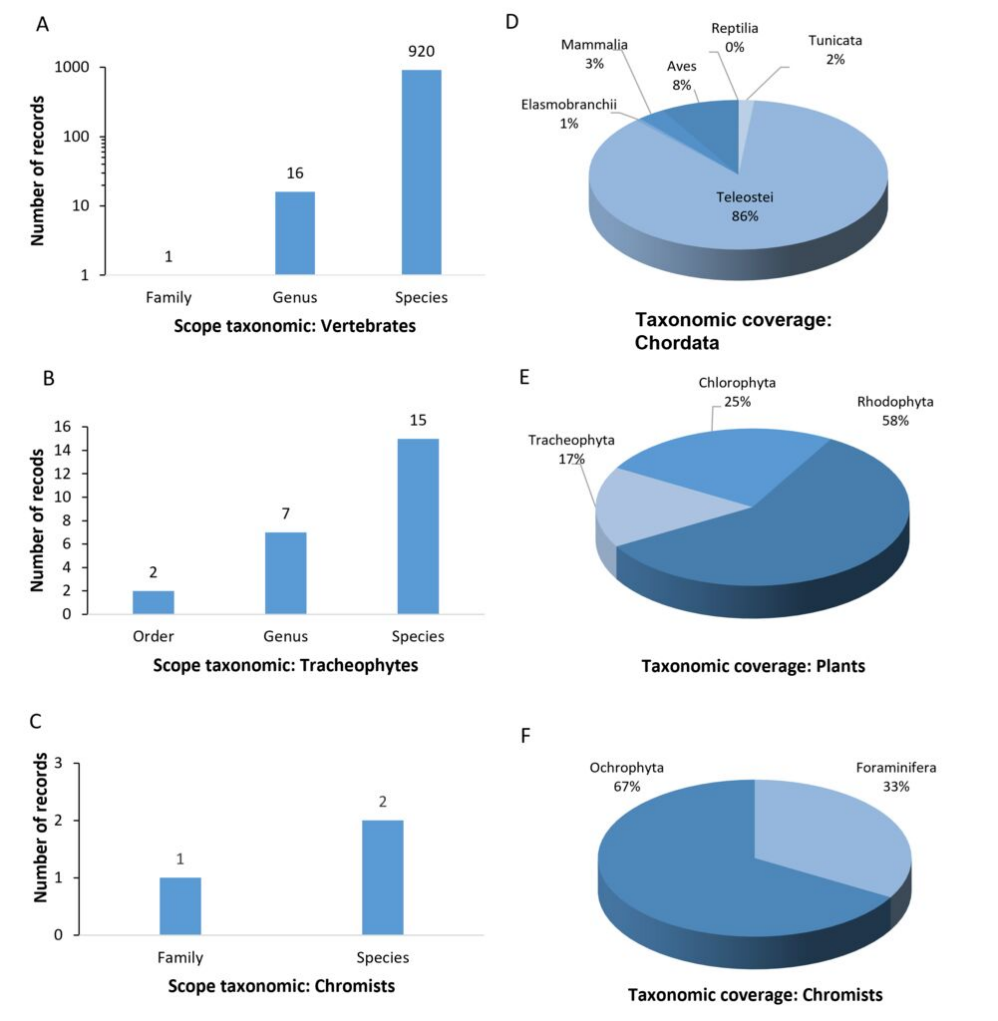
Details of the scope and taxonomic coverage reached. Left: Scope by taxonomic categories considering the total number of records for vertebrates (A), Trachephytes (B) and Chromists (C). Right: Taxonomic coverage by phyla considering only the specimens proporcion identified to species level for vertebrates (D), Trachephytes (E) and Chromists (F).

In terms of the scope of the general taxonomic identification, 77% of the specimens were identified at species level, 16% at genus level and the remaining 7% were assigned to higher categories (Fig. 2). The dominant phyla, ranked by number of records were as follows: Chordata (937 records), Mollusca (352) and Arthropoda (338) for Animalia. For Plantae: Rhodophyta (14 records). For Chromista: Ochrophyta (4 records). The details of the scope and general coverage riched for invertebrates are shown in Fig. 3 and for vertebrates, chromists and plants in Fig. 4.

## Temporal coverage

**Data range:** 2024-6-28 – 2024-8-10.

## Collection data

### Collection name

Museo de Historia Natural Marina de Colombia - MHNMC. Coleccion Hidrobiológica de la Universidad Tecnológica del Chocó - CHbCh. Colecciones marinas de referencia de Biología Marina de la Universidad del Valle: Colección de Equinodermos - CRBMeq-UV, Colección de Crustáceos - CERBMcr-UV, Colección de Corales - CRBMco, Colección de Anélidos - CRBM-UV, Colección de Moluscos - CRM-UV, Colección Ictiológica de Referencia Universidad del Valle (CIR-UV).

### Collection identifier

MHNMC | CHbCh | Marine collections of Universidad del Valle: CRBMeq-UV, CERBMcr-UV, CRBMco, CRBM-UV, CRM-UV, CIR-UV.

### Specimen preservation method

Alcohol 96%.

### Curatorial unit

Between 1 and 1,302 lots of specimens. Between 1 and 2,107 biological records.

## Usage licence

### Usage licence

Creative Commons Public Domain Waiver (CC-Zero)

## Data resources

### Data package title

Biodiversidad marina encontrada durante la expedición Colombia BIO Juradó-Cupica, departamento del Chocó, Pacífico norte colombiano - Proyecto Colombia BIO

### Resource link

https://doi.org/10.15472/kz6mh8 | https://obis.org/dataset/36ebf033-10b8-42a8-ad21-38b1b78d1867 | https://www.gbif.org/dataset/6e5801dd-296a-4318-95ce-64ea11e7efc8

### Alternative identifiers

https://ipt.biodiversidad.co/sibm/resource?r=biojurado-cupica | 36ebf033-10b8-42a8-ad21-38b1b78d1867 | 6e5801dd-296a-4318-95ce-64ea11e7efc8

### Number of data sets

2

### Data set 1.

#### Data set name

Occurrence Table

#### Data format

Darwin Core Archive (DwC-A) - Table data: TSV/TXT

#### Character set

UTF-8

#### Download URL

https://ipt.biodiversidad.co/sibm/resource?r=biojurado-cupica | https://obis.org/dataset/36ebf033-10b8-42a8-ad21-38b1b78d1867 | https://www.gbif.org/dataset/6e5801dd-296a-4318-95ce-64ea11e7efc8

#### Data format version

2.3

#### Description

The dataset compiles 2,107 biological records in an occurrence Darwin Core Archive (DwC-A) file, with 103 DwC terms documented. The OBIS-GBIF Integrated Publishing Toolkit (IPT) v.3.1.5 was used to facilitate the publication, with the process executed via the SiBM-OBIS Colombia IPT Portal. The dataset was also published on the Ocean Biodiversity Information System (OBIS), as well as on the Global Biodiversity Information Facility (GBIF) platforms (Dorado-Roncancio et al. 2025).

**Data set 1.** 

Column label	Column description
occurrenceID	An identifier for the Occurrence.
basisOfRecord	The specific nature of the data record.
type	The nature or genre of the resource.
institutionCode	The name (or acronym) in use by the institution having custody of the object(s) or information referred to in the record.
institutionID	An identifier for the institution having custody of the object(s) or information referred to in the record.
collectionCode	The acronym that identifying the collection or dataset from which the record was derived.
collectionID	An identifier for the collection or dataset from which the record was derived.
catalogNumber	A unique identifier for each the record within the dataset or collection.
datasetName	The name identifying the dataset from which the record was derived.
datasetID	An identifier for the set of data. May be a global unique identifier or an identifier specific to a collection or institution.
language	A language of the resource.
licence	A legal document giving official permission to do something with the resource.
rightsHolder	An organisation owning or managing rights over the resource.
accessRights	Information about who can access the resource or an indication of its security status.
recordNumber	An identifier given to the Occurrence at the time it was recorded. It serves as a link between field notes and an Occurrence record, such as a specimen collector's number.
recordedBy	A list (concatenated and separated) of names of people responsible for recording the original Occurrence. The primary collector or observer is listed first.
recordedByID	A list (concatenated and separated) of the globally unique identifier for the person responsible for recording the original Occurrence.
organismID	A specific identifier for the Organism instance (as opposed to a particular digital record of the Organism) in the dataset.
individualCount	The number of individuals present at the time of the Occurrence.
vitality	An indication of whether an Organism was alive or dead at the time of collection or observation.
behaviour	The behaviour shown by the subject at the time the Occurrence was recorded.
occurrenceStatus	A statement about the presence or absence of a Taxon at a Location.
preparations	A list (concatenated and separated) of preparations and preservation methods for a MaterialEntity.
disposition	The current state of a MaterialEntity with respect to a collection.
otherCatalogNumbers	A list (concatenated and separated) of alternative fully qualified catalogue numbers or other human-used identifiers for the same Occurrence.
associatedSequences	A list (concatenated and separated) of identifiers (publication, global unique identifier, URI) of genetic sequence information associated with the MaterialEntity.
occurrenceRemarks	Comments or notes about the Occurrence.
parentEventID	An identifier for the broader Event that groups this and potentially other Events.
eventID	A identifier for the set of information associated with an Event (something that occurs at a place and time). This is an identifier specific to the dataset.
eventType	The nature of the Event.
samplingProtocol	The names of, references to, or descriptions of the methods or protocols used during an Event.
sampleSizeValue	A numeric value for a measurement of the size (time duration, length, area or volume) of a sample in a sampling Event.
sampleSizeUnit	The unit of measurement of the size (time duration, length, area or volume) of a sample in a sampling Event.
samplingEffort	The amount of effort expended during an Event.
eventDate	The date-time or interval during which an Event occurred. For occurrences, this is the date-time when the Event was recorded.
year	The four-digit year in which the Event occurred, according to the Common Era Calendar.
month	The integer month in which the Event occurred.
day	The integer day of the month on which the Event occurred.
eventTime	The time or interval during which an Event occurred.
habitat	A category or description of the habitat in which the Event occurred.
fieldNumber	An identifier given to the Event in the field. It serves as a link between field notes and the Event.
fieldNotes	An indicator of the existence of the text of notes taken in the field about the Event.
eventRemarks	Comments or notes about the dwc:Event.
locationID	An identifier for the set of Location information. May be a global unique identifier or an identifier specific to the dataset.
continent	The name of the continent in which the Location occurs.
waterBody	The name of the waterbody in which the Location occurs.
country	The name of the country or major administrative unit in which the Location occurs.
countryCode	The standard code for the country in which the Location occurs.
stateProvince	The name of the next smaller administrative region than country (state, province, canton, department, region etc.) in which the Location occurs.
county	The full, unabbreviated name of the next smaller administrative region than stateProvince (county, shire, department etc.) in which the Location occurs.
municipality	The full, unabbreviated name of the next smaller administrative region than county (city, municipality etc.) in which the Location occurs. Do not use this term for a nearby named place that does not contain the actual Location.
locality	The specific description of the place.
verbatimLocality	The original textual description of the place.
minimumDepthInMetres	The lesser depth of a range of depth below the local surface, in metres.
maximumDepthInMetres	The greater depth of a range of depth below the local surface, in metres.
locationAccordingTo	Information about the source of this Location information. Could be a publication (gazetteer), institution or team of individuals.
locationRemarks	Comments or notes about the Location.
verbatimLatitude	The verbatim original latitude of the Location.
verbatimLongitude	The verbatim original longitude of the dcterms:Location.
verbatimCoordinateSystem	The coordinate format for the verbatimLatitude and verbatimLongitude or verbatimCoordinates of the Location.
verbatimSRS	The ellipsoid, geodetic datum or spatial reference system (SRS), upon which coordinates given in verbatimLatitude and verbatimLongitude or verbatimCoordinates are based.
decimalLatitude	The geographic latitude (in decimal degrees, using the spatial reference system given in geodeticDatum) of the geographic centre of a Location.
decimalLongitude	The geographic longitude (in decimal degrees, using the spatial reference system given in geodeticDatum) of the geographic centre of a Location.
geodeticDatum	The ellipsoid, geodetic datum or spatial reference system (SRS), upon which the geographic coordinates given in decimalLatitude and decimalLongitude are based.
footprintWKT	A Well-Known Text (WKT) representation of the shape (footprint, geometry) that defines the Location. A Location may have both a point-radius representation (see decimalLatitude) and a footprint representation, and they may differ from each other.
georeferencedBy	A list (concatenated and separated) of names of people who determined the georeference (spatial representation) for the Location.
georeferencedDate	The date on which the Location was georeferenced.
georeferenceProtocol	A description or reference to the methods used to determine the spatial footprint, coordinates and uncertainties.
georeferenceSources	A list (concatenated and separated) of maps, gazetteers or other resources used to georeference the Location, described specifically enough to allow anyone in the future to use the same resources.
georeferenceVerificationStatus	A categorical description of the extent to which the georeference has been verified to represent the best possible spatial description for the Location of the Occurrence.
georeferenceRemarks	Comments or notes about the spatial description determination, explaining assumptions made in addition or opposition to the those formalised in the method referred to georeferenceProtocol.
identifiedBy	A list (concatenated and separated) of names of people who assigned the Taxon to the subject.
identifiedByID	A list (concatenated and separated) of the globally unique identifier for the person responsible for assigning the Taxon to the subject.
dateIdentified	The date on which the subject was determined as representing the Taxon.
identificationReferences	A list (concatenated and separated) of references (publication, global unique identifier, URI) used in the Identification.
identificationVerificationStatus	A categorical indicator of the extent to which the taxonomic identification has been verified to be correct.
verbatimIdentification	A string representing the taxonomic identification as it appeared in the original record.
identificationRemarks	Comments or notes about the Identification.
identificationQualifier	A brief phrase or a standard term ("cf.", "aff.") to express the determiner's doubts about the Identification.
scientificName	The full scientific name, at the lowest level taxonomic rank that could be determined.
scientificNameAuthorship	The authorship information for the scientificName formatted according to the conventions of the applicable nomenclaturalCode.
taxonID	An unique identifier for the set of Taxon information.
scientificNameID	An identifier for the nomenclatural (not taxonomic) details of a scientific name.
kingdom	The full scientific name of the kingdom in which the Taxon is classified.
phylum	The full scientific name of the phylum or division in which the Taxon is classified.
class	The full scientific name of the class in which the Taxon is classified.
order	The full scientific name of the order in which the dwc:Taxon is classified.
superfamily	The full scientific name of the superfamily in which the Taxon is classified.
family	The full scientific name of the family in which the Taxon is classified.
subfamily	The full scientific name of the subfamily in which the Taxon is classified.
tribe	The full scientific name of the tribe in which the Taxon is classified.
genus	The full scientific name of the genus in which the Taxon is classified.
subgenus	The full scientific name of the subgenus in which the Taxon is classified.
specificEpithet	The name of the first or species epithet of the scientificName.
taxonRank	The taxonomic rank of the most specific name in the scientificName.
verbatimTaxonRank	The taxonomic rank of the most specific name in the scientificName as it appears in the original record.
vernacularName	A common or vernacular name.
taxonomicStatus	The status of the use of the scientificName as a label for a taxon.
nomenclaturalCode	The nomenclatural code (or codes in the case of an ambiregnal name) under which the scientificName is constructed.
taxonRemarks	Comments or notes about the taxon or name.

### Data set 2.

#### Data set name

Extended extension of Meassurements of Facts (eMoF) Table

#### Data format

Darwin Core Archive (DwC-A) - Table data: TXT/TSV

#### Character set

UTF-8

#### Download URL

https://ipt.biodiversidad.co/sibm/resource?r=biojurado-cupica | https://obis.org/dataset/36ebf033-10b8-42a8-ad21-38b1b78d1867 | https://www.gbif.org/dataset/6e5801dd-296a-4318-95ce-64ea11e7efc8

#### Data format version

2.3

#### Description

The eMoF table compiles 4,570 records for eight complementary variables (sampling equipment, individual counts, other types quantity, sex, life stages, behaviour and vitality), derived from biological records. The data were structured as a Darwin Core eMoF extension file, with eight DwC terms documented. This table complementing core Occurrence file in DwC-A. The OBIS-GBIF Integrated Publishing Toolkit (IPT) v.3.1.5 was used to facilitate the publication, with the process executed via the SiBM-OBIS Colombia IPT Portal. The dataset was also published on the Ocean Biodiversity Information System (OBIS), as well as on the Global Biodiversity Information Facility (GBIF) platforms (Dorado-Roncancio et al. 2025).

**Data set 2.** 

Column label	Column description
occurrenceID	The identifier of the occurrence the measurement or fact refers to.
measurementID	An identifier for the MeasurementOrFact (information pertaining to measurements, facts, characteristics or assertions). May be a global unique identifier or an identifier specific to the dataset.
measurementType	The nature of the measurement, fact, characteristic or assertion.
measurementTypeID	An identifier for the measurementType (global unique identifier, URI). The identifier should reference the measurementType in a vocabulary.
measurementValue	The value of the measurement, fact, characteristic or assertion.
measurementValueID	An identifier for facts stored in the column measurementValue (global unique identifier, URI). This identifier can reference a controlled vocabulary or reference a methodology paper with a DOI. When the measurementValue refers to a value and not to a fact, the measurementvalueID has no meaning and should remain empty.
measurementDeterminedDate	The date on which the MeasurementOrFact was made.
measurementRemarks	Comments or notes accompanying the MeasurementOrFact.

## Additional information

**New records and Distribution**

Expedition results: 2,107 records, revealing a considerable taxonomic richness with 96 new records for northern Chocó [fishes (39), annelids (32), cnidaria (7), porifera (6), tunicates (8), arthropods (2), bryozoans (2), chlorophytes (1)]. Forty-four new taxa for Colombian Pacific coast [annelids (20), cnidaria (7), porifera (6), tunicates (8), arthropods (2) and chlorophytes (1) are registered for first time; detailed in Table 1.

**Ecosystems biodiversity**

**Figure 5.**
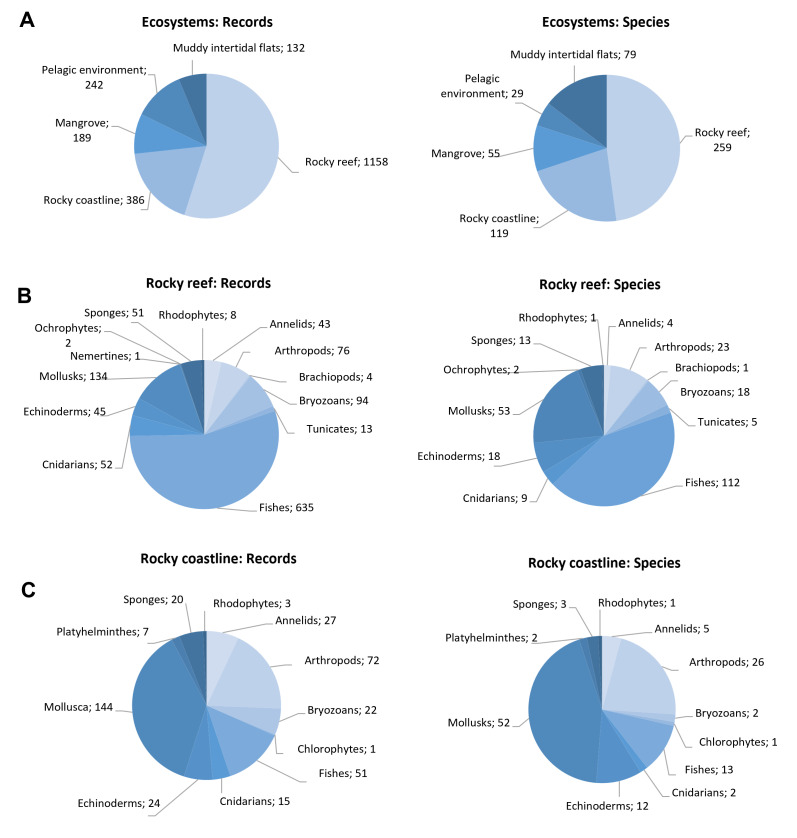
Species and morphospecies were found by ecosystem (A) and detailed on rocky reef (B) and rocky coastline (C).

**Figure 6.**
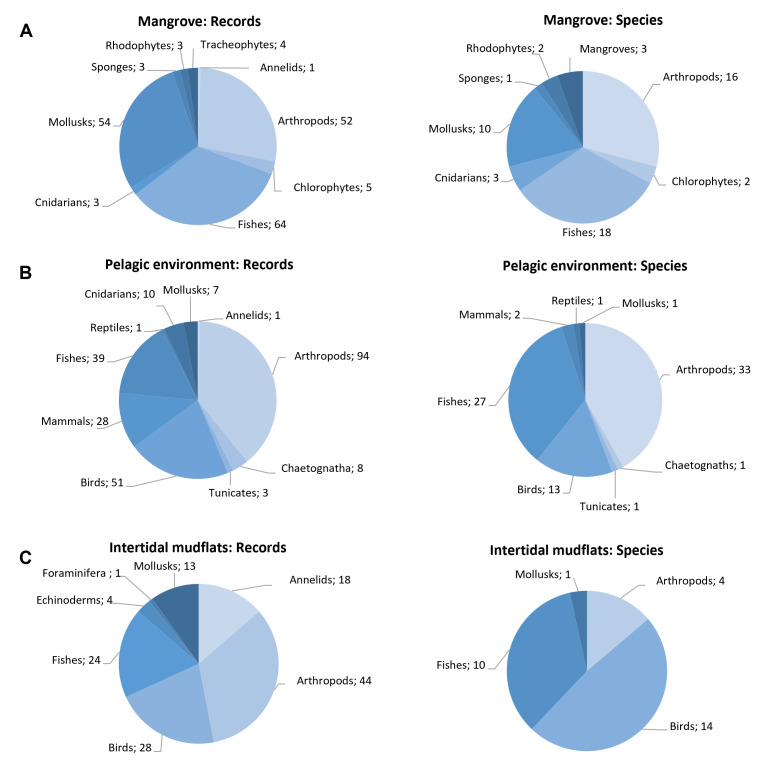
Species and morphospecies were found on mangroves (A), pelagic (B) and intertidal mudflats (C).

By ecosystems assessed, the highest number of species and morphospecies found were in mangroves, followed by rocky coastline, intertidal mudflats, rocky reefs and the pelagic environment (Fig. 5; Fig. 6). In terms of both species diversity and total occurrences recorded, the most representative groups were fish, molluscs and arthropods.

**Conservation implications**

The information here provided contributes to the baseline on marine biodiversity in a remote territory for science and in perfect timing due to recent designation of a biosphere reserve including part of the area we investigated on the expedition and in front of apparent return of historical interest on projects to connect the Caribbean Sea and Pacific Ocean through the Chocó-Darién Region. The number of species here reported is significant and contributes to fill the gap in TEP marine biodiversity notoriously located in the Colombian North Pacific. Hopefully, this results may be inputs to strength or discuss marine biogegraphical hypotesis in the TEP marine ecoregion. Besides, the results highlight the territory's environmental richness and its importance to the livelihoods of its inhabitants (specially fishermen), while also point out its vulnerability to political decisions regarding development trajectory best suited from an environmental and social perspective.

## Supplementary Material

D2C2D358-0A38-597D-AB5D-127217D398A0 10.3897/BDJ.14.e189764.suppl1 Supplementary material 1 Table_Site and Sampling Stations Data type stations Brief description Details of the sites and sampling stations that were visited during the 'Colombia BIO Juradó-Cupica Expedition' in 2024. File: oo_1547082.tsv https://binary.pensoft.net/file/1547082 Edgar Fernando Dorado-Roncancio, Erika Montoya-Cadavid, Armando Rodríguez-Toscano, Luis H. Chasqui

## Figures and Tables

**Table 1.** New species records found during the '*Colombia BIO Juradó-Cupica Expedition*' in the North Pacific of Chocó and the Colombian Pacific Region.

**Phylum**	**Family**	**Specie**	**New record for the study area**	**New record for the Colombian Pacific**
Annelida	Amphinomidae	*Eurythoe pacifica* Kinberg, 1857	X	X
		*Eurythoe* sp.	X	
	Antillesomatidae	*Antillesoma mexicanum* Silva-Morales, López-Aquino, Islas-Villanueva, Ruiz-Escobar & Bastida-Zavala, 2019	X	X
	Capitellidae	*Leiochrides* sp.	X	X
	Eunicidae	*Eunice* sp.	X	
		*Marphysa* sp.	X	
		*Palola* sp.1	X	
		*Palola* sp.2	X	
	Iphionidae	Iphione cf. muricata (Lamarck, 1818)	X	X
		*Iphione* sp.	X	X
	Nereididae	*Ceratonereis* sp.	X	X
		*Rullierinereis* sp.	X	X
		*Imajimainereis* sp.	X	X
	Phascolosomatidae	Phascolosoma (Phascolosoma) sp.	X	
		Phascolosoma (Phascolosoma) azteca Gómez-Vásquez, 2024	X	X
	Phyllodocidae	*Eulalia myriacyclum* (Schmarda, 1861)	X	X
	Polynoidae	*Thormora* sp.	X	
	Sabellariidae	*Idanthyrsus cretus* Chamberlin, 1919	X	
		*Phragmatopoma carlosi* Chávez-López, 2020	X	X
	Serpulidae	*Hydroides crucigera* Mörch, 1863	X	
		Pyrgopolon cf. ctenactis (Mörch, 1863)	X	
		*Spirobranchus* sp.1	X	
		*Spirobranchus* sp.2	X	
	Sigalionidae	*Pisione* sp.	X	X
	Spionidae	*Boccardia* sp.	X	X
	Syllidae	cf. *Trypanedenta* sp.	X	X
		*Odontosyllis* sp.	X	X
		*Exogone* sp.	X	X
	Terebellidae	*Amphitritides bruneocomata* (Ehlers, 1887)	X	X
		*Pista cetrata* (Ehlers, 1887)	X	X
		*Neoleprea* sp.	X	X
	Thalassematidae	*Anelassorhynchus* sp.	X	X
Arthropoda	Ocypodidae	*Leptuca tallanica* (von Hagen, 1968)	X	X
	Panopeidae	*Panopeus rugosus* A. Milne-Edwards, 1880	X	X
Bryozoa	Cupuladriidae	*Discoporella triangula*Herrera-Cubilla, Dick, Sanner & Jackson, 2008	X	
	Mamilloporidae	*Mamillopora cupula* Smitt, 1873	X	
Cnidaria	Actiniidae	*Anthopleura nigrescens* (Verrill, 1928)	X	X
	Andvakiidae	*Telmatactis* sp.1	X	X
		*Telmatactis* sp.2	X	X
		*Telmatactis* sp.3	X	X
		*Telmatactis* sp.4	X	X
	Campanulariidae	*Clytia elsaeoswaldae* Stechow, 1914	X	X
		*Obelia bidentata* Clark, 1875	X	X
Porifera	Aplysinidae	*Aplysina clathrata* Cruz-Barraza, Carballo, Rocha-Olivares, Ehrlich & Hog, 2012	X	X
	Chalinidae	Haliclona (Gellius) perforata (Wilson, 1904)	X	X
	Chondrillidae	*Chondrilla pacifica* Carballo, Gómez, Cruz-Barraza & Flores-Sanchez, 2003	X	X
	Hymedesmiidae	*Acanthancora equiformis* Sim-Smith, Hickman & Kelly, 2021	X	X
	Mycalidae	Mycale (Carmia) cecilia de Laubenfels, 1936	X	X
	Timeidae	*Timea floridusa* Carballo & Cruz-Barraza, 2006	X	X
Chordata	Ascidiidae	*Ascidia sideralis* Bonnet & Rocha, 2013	X	X
		*Ascidia* sp.1	X	X
		*Ascidia* sp.2	X	X
	Diazonidae	*Rhopalaea birkelandi* Tokioka, 1971	X	X
	Didemnidae	*Polysyncraton* sp.	X	X
	Pyuridae	*Pyura carmanae* (Jordan & Gilbert, 1882)	X	X
	Styelidae	*Symplegma brakenhielmi* (Michaelsen, 1904)	X	X
		*Symplegma rubra* Monniot C., 1972	X	X
	Acanthuridae	*Acanthurus triostegus* (Linnaeus 1758)	X	
	Belonidae	*Tylosurus pacificus* (Steindachner, 1876)	X	
	Carangidae	*Caranx vinctus* Jordan & Gilbert, 1882	X	
		*Selar crumenophthalmus* (Bloch 1793)	X	
		*Selene peruviana* (Guichenot 1866)	X	
	Centropomidae	*Centropomus armatus* Gill, 1863	X	
		*Centropomus medius* Günther, 1864	X	
	Chaenopsidae	*Acanthemblemaria balanorum* Brock, 1940	X	
		*Coralliozetus springeri* Stephens & Johnson, 1966	X	
		*Protemblemaria bicirrus* (Hildebrand, 1946)	X	
	Congridae	*Heteroconger klausewitzi* Eibl-Eibesfeldt & Köster, 1983	X	
	Eleotridae	*Erotelis armiger* (Jordan & Richardson, 1895)	X	
		Gobiomorus cf. polylepis	X	
	Gerreidae	*Deckertichthys aureolus* (Jordan & Gilbert 1882)	X	
		*Eucinostomus currani* Zahuranec, 1980	X	
	Gobiesocidae	Gobiesox cf. woodsi (Schultz, 1944)	X	
		Tomicodon cf. zebra (Jordan & Gilbert, 1882)	X	
	Gobiidae	*Awaous transandeanus* (Gunther, 1861)	X	
		*Bathygobius andrei* (Sauvage, 1880)	X	
		*Ctenogobius sagittula* (Günther, 1861)	X	
		*Evorthodus minutus* Meek & Hildebrand, 1928	X	
		*Tigrigobius inornatus* Bussing, 1990	X	
	Haemulidae	*Haemulopsis axillaris* (Steindachner, 1869)	X	
	Labridae	*Halichoeres adustus* (Gilbert, 1890)	X	
	Labrisomidae	*Malacoctenus sudensis* Springer, 1959	X	
	Lutjanidae	*Lutjanus colorado* (Jordan & Gilbert, 1882)	X	
	Mugilidae	*Mugil setosus* Gilbert, 1892	X	
	Muraenesocidae	*Cynoponticus coniceps* (Jordan & Gilbert 1882)	X	
	Paralichthyidae	*Citharichthys gilberti* Jenkins & Evermann, 1889	X	
		*Cyclopsetta querna* (Jordan & Bollman 1890)	X	
	Poeciliidae	*Priapichthys panamensis* Meek & Hildebrand, 1916	X	
	Polynemidae	*Polydactylus approximans* (Lay & Bennett 1839)	X	
	Sciaenidae	*Cynoscion phoxocephalus* Jordan & Gilbert 1882	X	
		*Umbrina analis* Günther, 1868	X	
		*Umbrina xanti* Gill, 1862	X	
	Scombridae	*Katsuwonus pelamis* (Linnaeus, 1758)	X	
	Scorpaenidae	*Scorpaenodes xyris* (Jordan & Gilbert, 1882)	X	
	Tetraodontidae	*Sphoeroides annulatus* (Jenyns, 1842)	X	
Chlorophyta	Caulerpaceae	*Caulerpa verticillata* J.Agardh, 1847	X	X
